# Anetoderma may be a warning sign of autoimmunity: A cohort study

**DOI:** 10.1016/j.jdin.2024.02.002

**Published:** 2024-02-13

**Authors:** Shawn Afvari, Rhea Malik, Baraa Hijaz, Vinod E. Nambudiri

**Affiliations:** a Department of Dermatology, Brigham and Women’s Hospital, Boston, Massachusetts; b New York Medical College School of Medicine, Valhalla, New York; c Harvard Medical School, Boston, Massachusetts

**Keywords:** anetoderma, atrophic skin, autoimmune, histopathology, rheumatoid arthritis, Sjogren syndrome, systemic lupus erythematosus

*To the Editor:* Anetoderma—an uncommon atrophic skin disorder—presents clinically with flesh-colored atrophic depressions and saccular protrusions and is marked histopathologically by dermal elastolysis. While lesions of anetoderma are widely considered benign,^1^^,^^2^ case reports have associated its incidence with systemic autoimmune conditions.^3^, ^4^, ^5^ We performed a retrospective cohort study to assess clinical presentations of anetoderma and associated diagnoses and comorbidities.

We queried the Research Patient Data Registry at Mass General Brigham from January 1980 to August 2022 with the search term “anetoderma” in all pathology reports. Patients with confirmed anetoderma were defined as those with “anetoderma” as the favored diagnosis on both the histopathological and examining clinician’s report. Patients who underwent biopsy for suspected anetoderma but ultimately did not have pathological diagnosis served as our comparator group. Patients with biopsies demonstrating some histopathologic features consistent with anetoderma but without clinical correlation were excluded from our analysis. This analysis focused on patients with primary anetoderma (appearing on previously normal skin) and was self-reported, consistent with initial disease presentations. We recorded data on the Research Electronic Data Capture platform and performed all statistical analysis using JASP Minitab 15.1.0.0 software. The Mass General Brigham IRB approved this study.

Our search yielded 121 individuals, of whom 31 had a clinicopathologically-correlative diagnosis of confirmed anetoderma. These individuals were predominantly female (67.7%) and White (82.8%), with a mean age at biopsy of 44.1 (Table I). Lesions were most commonly located on the abdomen (33.3%), arms (20.0%), and neck (16.7%). Anetoderma lesions were most generally asymptomatic (60.0%); pruritus (32.0%) or pain (12.0%) were associated with a minority of lesions (Table II).

**Table I:** Patient demographics, diagnostic, and clinical associations

	Clinicopathological diagnosis
	(*N* = 31) *N* (%)
Sex	
Female, *N* (%)	21 (67.7)
Male, *N* (%)	10 (32.3)
Race/ethnicity, *N* (%)	
White	24 (77.4)
Hispanic	3 (9.7)
Asian	1 (3.2)
Black	1 (3.2)
Mean age at biopsy, years	44.1 (range: 17-75)

Time frame of first ANA positivity	(*N* = 12) *N* (%)
Preceding anetoderma diagnosis	6 (50.0)
Range	32.5 d-17.0 y
Mean	9.6 y
Median	10.5 y
Following anetoderma diagnosis	6 (50.0)
Range	1.2 d-13.7 y
Mean	4.6 y
Median	1.5 y

Time frame of first systemic autoimmune disorder development	(*N* = 9) *N* (%)
Preceding anetoderma diagnosis	5 (55.6)
Range	277 d-14.2 y
Mean	8.0 y
Median	7.8 y
Following anetoderma diagnosis	4 (44.4)
Range	173 d-7.7 y
Mean	3.8 y
Median	3.5 y

*ANA*, Antinuclear antibody.

**Table II:** Clinical presentation

	Clinicopathological diagnosis
	(*N* = 31) *N* (%)
Distribution∗	(*N* = 30) *n* (%)
Trunk	15 (50.0)
Upper extremity	10 (33.3)
Lower extremity	5 (16.7)
Neck	5 (16.7)
Back	4 (13.3)
Symptoms∗	(*N* = 25) *n* (%)
Asymptomatic	15 (60.0)
Itch	8 (32.0)
Pain/burning	5 (20.0)
Exam findings∗	(*N* = 12) *n* (%)
Epidermal atrophy	6 (50.0)
Erythema	3 (25.0)
Cigarette paper	2 (16.7)
Laxity	2 (16.7)
Outpouching	3 (25.0)
Color change	(*N* = 15) *n* (%)
Skin color	6 (40.0)
White	6 (40.0)
Pink	3 (20.0)
Number of lesions	(*N* = 22) *n* (%)
Single	11 (50.0)
Numerous	11 (50.0)

∗ Examining clinicians may have listed more than one descriptor for a single patient.

The comparator group consists of 78 patients whose lesions were not consistent with anetoderma on histopathological diagnosis. Patients with anetoderma were significantly more likely to have higher antinuclear antibody titers and overall antinuclear antibody positivity than those without anetoderma (*P* value = .025). A systemic autoimmune disorder was diagnosed in 29.0% of patients (*n* = 9), with rheumatoid arthritis and Sjogren syndrome as the most common comorbidities. Patients with anetoderma were significantly more likely to have or develop a systemic autoimmune condition than those without anetoderma (*P* value = .044). There was no significant difference between groups in autoimmune skin conditions (*P* value = .137).

Our results highlight associations between anetoderma incidence and autoimmunity, which was previously limited to case reports.^3^, ^4^, ^5^ Of note, we did not find any significant incidence of other major comorbidities such as heart failure, diabetes, renal disease, or liver disease in our anetoderma cohort to provide meaningful comment.

An anetoderma lesion should be seen as a possible sign of autoimmunity, and dermatologists should consider screening patients with new anetoderma diagnosis for underlying autoimmune conditions or comorbidities. Limitations of our study include its retrospective nature across 2 academic centers and our limited insight into the clinical characteristics of the anetoderma lesions that are deemed clinically necessary to biopsy. We hope this study offers new insight into this condition and underscores the need for laboratory evaluation of associated comorbidities in patients presenting with anetoderma.

## Conflicts of interest

None disclosed.

## References

[1] Cook J.C., Puckett Y. (2022). Anetoderma. StatPearls. StatPearls Publishing.

[2] Persechino S., Caperchi C., Cortesi G. (2011). Anetoderma: evidence of the relationship with autoimmune disease and a possible role of macrophages in the etiopathogenesis. Int J Immunopathol Pharmacol.

[3] Stephansson E.A., Niemi K.M., Jouhikainen T., Vaarala O., Palosuo T. (1991). Lupus anticoagulant and the skin. A longterm follow-up study of SLE patients with special reference to histopathological findings. Acta Derm Venereol.

[4] Romaní J., Pérez F., Llobet M., Planagumá M., Pujol R.M. (2001). Anetoderma associated with antiphospholipid antibodies: case report and review of the literature. J Eur Acad Dermatol Venereol.

[5] Yélamos O., Barnadas M.A., Díaz C., Puig L. (2014). Primary anetoderma associated with primary Sjögren syndrome and anticardiolipin antibodies. Actas Dermosifiliogr.

